# On the Result of the Cæsarean Section

**Published:** 1842-07

**Authors:** 


### Art. XVIII.

*De Eventu Sectionis Cæsareæ, etc.* Auctore C. Kayser, Licentiato Medico.—*Havniæ*, 1841. 8vo, pp. 122.

*On the Result of the Cæsarean Section.* By C. Kayser.—*Copenhagen.* 1841.

It happens but rarely that we meet with inaugural dissertations of merit sufficient to claim a notice in these pages. For the most part they contain little that is either novel or important, but present hasty generalizations from a few cases, or consist of ill-arranged compilations made without any critical sagacity. It is but justice, however, to the University of Copenhagen to state, that a considerable proportion of the theses emanating from it are of a very different character, and of this number is that now before us. It is, indeed, a work which does honour at once to the author and to the university of which he is a member.

M. Kayser has applied himself with diligence to the investigation of a subject which required to be further elucidated than it has been hitherto, and with reference to which there is in this country especial need of more extended knowledge. No space is occupied with curious though profitless antiquarian discussions as to the origin of the name by which the operation is usually distinguished, or as to its performance among the nations of antiquity, but the author at once proceeds to matters of practical importance. His object is to present a list as complete as possible of all well-authenticated cases in which the Cæsarean section has been performed, to display the results of the operation, and to examine the causes to which its success or failure may be attributed. It is true that this task is by no means new, but it has never been executed so well as by M. Kayser. All of the older writers on the subject have fallen into the error of reporting cases which were ill substantiated, a fault which resulted in great measure from their object being not so much to arrive at a knowledge of the actual results of the operation, as to prove that it might be performed with success. Thus, the work of Rousset, published in 1581, which was the first apology for the Cæsarian section, contains twenty cases, but not one of these was observed by Rousset himself, while some might be rejected without hesitation as being evidently fabulous. The same fault attaches more or less to all writings on the subject down to the year 1750, since which time more attention has been paid to collect unsuccessful as well as successful cases. It is therefore with great propriety that M. Kayser divides the history of the Cæsarean section into two periods, of which the former terminates with the appearance of Simon's essay in 1749; and the latter extends from the year 1750 to the present day.

It will be seen by referring to Baudelocque's essay on the Cæsarean Section, or to its translation by Dr. Hull, that the commonly-received estimate of the results of the operation is based in a great measure on cases reported to have occurred before the year 1750. The same observation holds good with reference to the elaborate collection of Michaelis; and several of the 258 cases which he has reported rest on very slender authority. M. Kayser's calculations are founded on the reports of 338 cases in which the operation was performed subsequently to the year 1750; and he rejects all instances of its performance before that time, either as being insufficiently authenticated, or at least too imperfectly reported to admit of any useful deductions being made from them.

Of these 338 cases, occurring between 1750 and 1839, 128 had a fortunate result, as far as the mother's life was concerned, while 210 terminated fatally. These figures give us a mortality of 62 per cent., which is 10 per cent. higher than that deduced from the investigations of Michaelis, or Levy, a recent Danish writer,* and 23 per cent. higher than the estimate formed by Dr. Hull. It appears, too, very probable that many cases in which the operation was performed have never been reported; and this was especially likely to be the case in former times, when medical journals were less numerous than they are at present. Unsuccessful cases were more likely to be left unreported than those in which the operation had a fortunate result, and it is therefore very probable that the real mortality is much higher than 62 or even than 67 per

* Dr. Churchill, in his elaborate work on Operative Midwifery, enumerates 316 operations between 1750 and 1841. In 149 cases the mother recovered, and 129 out of 182 children were saved: numbers from which results a mortality of 52·8 per cent. for the mothers, and of 29·8 per cent. for the children. Dr. Churchill's conclusions, therefore, tally exactly with those of Michaelis and Levy.

cent., as estimated by Velpeau. Wilde, indeed in his work entitled *Das weibliche Gebärunvermögen*, calculated the mortality at 90 per cent., and M. Kayser hesitates to assert positively that that estimate is too high.

Leaving these suppositions, however, and adopting the data with which M. Kayser has furnished us, we arrive at one very encouraging result; namely, that the fatality from the operation has been progressively diminishing since the year 1750.

| | Successful cases. | Fatal cases. | Rate of mortality. |
|---|---|---|---|
| 1750 to 1800 | 37 | 80 | 68 per cent. |
| 1801 1832 | 54 | 94 | 63 |
| 1833 1839 | 37 | 36 | 49 |
| | 128 | 210 | (p. 99.) |

The event, as regards the life of the child, has not been noted in all cases, but it does not seem that the mortality has undergone any important diminution, but has continued with occasional slight fluctuations at about 31 per cent.

After ascertaining the absolute mortality of mother and child from the Cæsarean section, M. Kayser next passes to the enquiry, (p. 109,) whether there exists any connexion between the recovery of the mother and the survival of the child. Michaelis denied the existence of any such connexion. It results, however, from our author's investigations, that only 27 per cent. of the infants were still-born in cases where the mothers did well, while their mortality amounted to 32 per cent. in those instances in which the mother lost her life.

The chief points which might be expected to influence the event of the operation both to mother and child, are the duration of the labour, the length of the interval between the rupture of the membranes and the performance of the Cæsarean section, and the circumstance of attempts having or not having been previously made to deliver the patient with the forceps. In cases where labour had lasted more than seventy-two hours, the mortality was 72 per cent. (p. 111;) in those where it had continued for a shorter time, only 61 per cent. The prospect of a favorable result for the mother is likewise increased by the performance of the operation before the rupture of the membranes, while the probabilities of an opposite result are greater when the liquor amnii has escaped long before the commencement of the operation. The influence of the duration of labour upon the infant's life, however, is much more striking. Sixty per cent. died when labour had lasted more than seventy-two hours, 33 per cent. when it had lasted between twenty-five and seventy-two hours, and only 28 per cent. when the Cæsarean section was performed within twenty-four after labour had begun. The influence of the escape of the liquor amnii upon the mortality of the infants is precisely such as we should anticipate. The numbers, 14, 22, and 49 per cent., representing the rate of their mortality according as the operation was performed within six or twenty-four hours after the rupture of the membranes, or after the lapse of a still longer time. The previous employment of instruments does not appear to have much influence on the fate of the mother, though it greatly diminishes the chances of survival for the child.

The supervention of inflammation is the most to be dreaded of all the consequences of the Cæsarean operation.

"In 123 cases the cause of death was stated with more or less accuracy; and it appears that 77 women died from inflammation, or its consequences, and 29 from the shock to the nervous system. Internal hemorrhage occurred in 10, in whom coagula of blood were found in the abdomen; 2 died from external hemorrhage; 2 from pneumonia; 1 from rupture of the uterus, and consequent hemorrhage, on the seventh day after delivery; 1 died from osteomalacia; and 1 from the immediate effects of the operation, only twenty-four hours after its completion." (p. 129.)

Inflammation, then, was the cause of death, in 63 per cent. of the cases, while only 24 per cent. died from the shock to the nervous system.

The date of the death of these persons was as follows: 1 died, as above noticed, immediately after the operation, 9 within six hours, 16 between six and twenty-four hours, and 108 within the first week, 16 died between the first and third week, 1 on the thirtieth, and 1 on the thirty-sixth day.

The above are only some of the principal points connected with the Cæsarean section which M. Kayser's diligence has elucidated. The influence of many other circumstances on the results of the operation is displayed; but we have reached the limits available for the present notice, and must refer our readers to the dissertation itself, of which we can honestly say, that the library of no teacher of obstetric medicine will be complete without it.

